# Antiseizure medication use during pregnancy and risk of ASD and ADHD in children

**DOI:** 10.1212/WNL.0000000000010993

**Published:** 2020-12-15

**Authors:** Kelsey K. Wiggs, Martin E. Rickert, Ayesha C. Sujan, Patrick D. Quinn, Henrik Larsson, Paul Lichtenstein, A. Sara Oberg, Brian M. D'Onofrio

**Affiliations:** From the Department of Psychological & Brain Sciences (K.K.W., M.E.R., A.C.S., B.M.D.) and Department of Applied Health Science (P.D.Q.), School of Public Health, Indiana University–Bloomington; Department of Medical Epidemiology and Biostatistics (H.L., P.L., A.S.O., B.M.D.), Karolinska Institutet, Stockholm; School of Medical Sciences (H.L.), Örebro University, Sweden; and Department of Epidemiology (A.S.O.), T.H. Chan School of Public Health, Harvard, Boston, MA.

## Abstract

**Objective:**

To determine whether children born to women who use antiseizure medications (ASMs) during pregnancy have higher risk of autism spectrum disorder (ASD) and attention-deficit/hyperactivity disorder (ADHD) independent of confounding factors.

**Methods:**

We used Swedish register data (n = 14,614 children born 1996–2011 and followed up through 2013) to examine associations in children of women with epilepsy, using the largest sample to date and adjusting for a range of measured confounders. We examined maternal-reported first-trimester use of any ASM (22.7%) and the 3 most commonly reported individual drugs (valproic acid 4.8%, lamotrigine 6.8%, and carbamazepine 9.7%). We identified ASD with ICD-10 diagnoses and ADHD with ICD-10 diagnoses or filled prescriptions of ADHD medication.

**Results:**

Examination of individual drugs revealed that after adjustment for confounding, use of valproic acid was associated with ASD (hazard ratio [HR] 2.30, 95% confidence interval [CI] 1.53–3.47) and ADHD (HR 1.74, 95% CI 1.28–2.38). Whereas a small, nonstatistically significant association with ASD (HR 1.25, 95% CI = 0.88–1.79) and ADHD (HR 1.18, 95% CI 0.91–1.52) remained for reported use of carbamazepine, confounding explained all of the associations with lamotrigine (HR_ASD_ 0.86, 95% CI 0.67–1.53; HR_ADHD_ 1.01, 95% CI 0.67–1.53).

**Conclusions:**

We found no evidence of risk related to exposure to lamotrigine, whereas we observed elevated risk of ASD and ADHD related to maternal use of valproic acid. Associations with carbamazepine were weak and not statistically significant. Our findings add to a growing body of evidence that suggests that certain ASMs may be safer than others in pregnancy.

Approximately 3 to 7 of every 1,000 pregnant women have epilepsy.^1,2^ The primary treatment is pharmacotherapy with antiseizure medications (ASMs).^3^ Concerns about ASM safety in pregnancy have been raised because research has shown risk of adverse birth outcomes^4^ and neurodevelopmental disorders (NDDs) such as autism spectrum disorder (ASD) and attention-deficit/hyperactivity disorder (ADHD)^5–11^ in exposed children. Research demonstrating that ASMs cross the human placenta^12–14^ and can result in aberrant neuronal development in rodents^15–17^ provides biological plausibility for a causal explanation of associations.

However, the use of small samples has limited the ability of previous studies to differentiate observed associations from the null hypothesis,^5–9^ leading to discrepant findings.^5–8,10,11^ Noncausal explanations (e.g., maternal epilepsy, shared genetic and environmental risk factors for seizures and NDDs) have not been ruled out.^3,4,18–24^ The majority of research does not adjust for many, if any, confounding factors (e.g., adjustment for and severity of maternal epilepsy).^5,7–11^ In fact, recent reviews have called for research using larger samples and better confounder adjustment to disentangle potential causal effects from noncausal explanations.^4,18,25–27^

The current study examined associations between maternal use of ASMs during pregnancy and childhood risk of ASD and ADHD using data from linked Swedish registers. This study adds to the previous literature by using the largest known sample to date and considering many confounders not accounted for in previous research. We examined associations among children born to women with epilepsy who did and did not use ASMs in pregnancy. We considered exposure to any ASM, as well as the 3 most commonly used ASMs in our sample (valproate, lamotrigine, and carbamazepine).

## Methods

### Standard protocol approvals, registrations, and patient consents

The institutional review board at Indiana University and the regional ethical review board in Stockholm, Sweden, approved this study. Informed consent was not necessary according to Swedish law because the study used data available from national registries.

### Data availability

The data used in this study are national register information made under ethical permission. The authors had no special privileges in accessing the data. Dissemination of personal information is regulated by the Swedish Secrecy Act. In accordance with Swedish law, researchers seeking access to individual-level data must apply for permission through a Research Ethics Board (etikprovningsmyndigheten.se) and from the primary owners, Statistics Sweden (scb.se/en/services/guidance-for-researchers-and-universities) and the National Board of Health and Welfare (socialstyrelsen.se/en/statistics-and-data/statistics/). Data available from Dryad (tables e-1 to e-3, doi.org/10.5061/dryad.2z34tmpk0).

### Data source

Each individual in Sweden is assigned a unique registration number that enables the linkage of information from national data registers and the following of events recorded in these registers across time. We used information from the Medical Birth Register, which contains information on >98% of births since 1973, including data from all antenatal visits, the delivery, and pediatric examination.^28,29^ The first antenatal visit typically occurs between the 8th and 12th week of pregnancy. In the standardized enrollment interview, midwives collect information on mothers' age, cohabitation status, reproductive history, and use of tobacco. Since mid-1995, women have also been asked about their prescription drug use. The Multi-Generation Register links individuals to their biological parents.^30^ We used the National Patient Register (NPR) for records of ICD-based diagnoses made during all inpatient care since 1987 and specialist outpatient visits since 2001.^31^ The Prescribed Drug Register provided information about prescription medications dispensed since July 2005.^32^ We used the Integrated Database for Labor Market Research^33^ and the Education Register for information on several socioeconomic factors.

### Sample

This study was based on records of children born to women with epilepsy any time before childbirth. We identified children born in Sweden between January 1, 1996, and December 31, 2011, and followed them up through December 31, 2013. We reduced our initial sample (n = 16,888) by sequentially dropping children who had missing paternal identifiers (n = 198), were multiples (n = 469), or had missing gestational age (n = 16). Table e-1 (doi.org/10.5061/dryad.2z34tmpk0) presents the distribution of relevant covariates, stratified by exposure, in our target cohort (i.e., including those with missing information), with exclusions applied. Further restriction of those with missing information on any of the relevant covariates (n = 1,591) led to a final analytic sample of 14,614 children (90.2% of target cohort).

### Measures

#### Exposures

We defined exposure using Anatomical Therapeutic Chemical (ATC) codes for maternal-reported ASM use (i.e., codes beginning with N03, plus N05BA09 [clobazam]) at the first antenatal visit. We identified use of any ASM (n = 3,316, 22.7%) and among them further found valproic acid (N03AG01; n = 699, 4.8%), lamotrigine (N03AX09; n = 996, 6.8%), and carbamazepine (N03AF01; n = 1,417, 9.7%) to be the most common, although prevalences changed over time. We present more information on the reported use of each individual drug over time in the figure.

**Figure:**
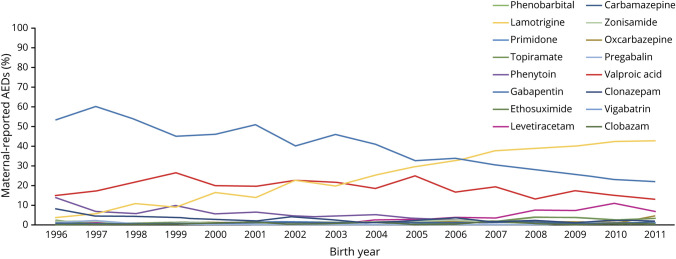
AED use over time AED = antiepileptic drug.

First, we cross-referenced maternal self-reports of ASMs against official records of filled prescriptions. This preliminary analysis included all births between 2006 and 2011 (n = 564,972) and verified previous findings of high agreement (κ = 0.7 for self-report and filled prescriptions in first trimester).^34^ Most maternal reports of ASMs (n = 1,658, 90%) were verified by filled prescriptions when we restricted exposure windows to the first trimester, and even more were verified (n = 1,729, 94%) when we examined filled prescriptions throughout pregnancy. This agreement provides support for our use of maternal report to define our exposure.

Next, we checked whether first-trimester reports were predictive of filled prescriptions of ASMs later in pregnancy. We examined this because, given that the majority of brain development occurs later in pregnancy, this is arguably a more critical window for insults to lead to neurodevelopmental dysfunction.^35–37^ However, because epilepsy is chronic and treatment discontinuation would likely be problematic for both pregnant women and fetal development, it would be expected that women who have chosen to maintain treatment in the first trimester would continue throughout the pregnancy. Among the women reporting use in the first trimester, 1,419 (77%) were found to have filled prescriptions later in pregnancy (second or third trimester), confirming expectations of continued use throughout pregnancy.

#### Outcomes

We defined ASD in children with inpatient and outpatient diagnoses made by specialists (recorded in the NPR using ICD-10 codes F84.0, F84.1, F84.5; n = 15,885, 1.06%). We defined ADHD (38,549, 2.57%) by either specialist diagnoses recorded in the NPR (ICD-10 code F90) or filled prescriptions for stimulant medication (ATC codes N06BA01, N06BA02, N06BA4, and N06BA09) recorded in the Prescribed Drug Register. For both outcomes, we considered only diagnoses made after 2 years of age. Children who were diagnosed with both ASD and ADHD (n exposed to any ASM = 52, 1.6%; n unexposed = 97, 0.9%) were included in our analyses. Our research group^38,39^ and others^40^ have shown that the ASD and ADHD diagnoses in the Swedish registers have high validity. In table 1, we present the number of cases and cumulative incidence of ASD and ADHD at age 10 for each exposure group. We note that the overall cumulative incidence of ASD (0.89%, n = 7,846) and ADHD (2.14%, n = 16,478) in the general population is lower than that of children in this sample born to women with epilepsy who did and did not use ASMs in pregnancy.

**Table 1:**
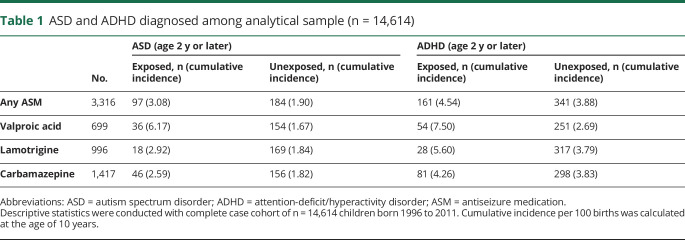
ASD and ADHD diagnosed among analytical sample (n = 14,614)

#### Maternal epilepsy

We identified maternal epilepsy using (1) any ICD-9/10 diagnosis (345 and G40, respectively, excluding infantile spasms: ICD-9 code 345.6) recorded in the NPR before childbirth or (2) a checkbox indicator or ICD-9/10 diagnosis recorded in the Medical Birth Register.

#### Other covariates

We first considered all known or hypothesized common causes of the exposure and outcomes to then select covariates that could block their influence (i.e., confounder adjustment; covariates presented in table e-1, doi.org/10.5061/dryad.2z34tmpk0). Maternal and paternal characteristics at the time the child was born included age, highest education, cohabitation status, and country of origin. We also included a measure of neighborhood deprivation (derived from a principal components analysis of yearly indicators for geographic areas)^41^ and averaged maternal and paternal disposable income in the year of birth as measures of socioeconomic factors. We further considered parental psychiatric and behavioral problems diagnosed before pregnancy using ICD-9 and ICD-10 codes (see table e-2, doi.org/10.5061/dryad.2z34tmpk0 for specific codes). We included any inpatient or outpatient diagnosis of bipolar disorder because this is an alternative indication for several ASMs, including valproic acid, lamotrigine, and carbamazepine. We also included suicide attempt, schizophrenia diagnosis, substance use disorder, and criminal convictions. We also adjusted for inpatient diagnosis of seizures in the year before pregnancy to capture and adjust for indication severity. Pregnancy-related characteristics included the year of birth, birth order, child sex, and maternal-reported smoking during pregnancy. For concurrent medication use, we considered maternal reports of other psychotropic medications, including lithium (ATC code N05AN01) antidepressants (ATC codes N06A), anxiolytics and sedatives (ATC codes N05B, N05C), antipsychotics (other ATC codes for N05A), ADHD medications (ATC codes N06BA01, N06BA02, N06BA04, N06BA09), and analgesics (i.e., opioids, acetaminophen, nonsteroidal anti-inflammatory drugs; ATC codes N02A, N02BE01, M01A).

### Data analytical plan

We estimated associations between exposure and outcomes using Cox proportional hazard regression models with attained age (from 2 years to first diagnosis or censoring) as the underlying time scale. Children were censored at time of emigration, death, or end of follow-up. All models compared maternal ASM use to no maternal ASM use in children born to women with epilepsy (i.e., for both any and specific ASMs, the comparison group was those with no report of ASM use). The sequence of models estimated was as follows. First, we obtained unadjusted estimates for the association between maternal reports of any AMS and ASD and ADHD, as well as separate estimates for the 3 most commonly reported drugs (valproic acid, lamotrigine, and carbamazepine). Second, we re-estimated the models adjusting for all time-independent covariates. Third, we fit the models with restriction to mothers who reported use of only 1 type of ASM (monotherapy). We did this because (1) some drug combinations may be more common and thus estimates may be confounded by individual effects of other drugs, (2) it is possible that drug-drug interactions^13,14,19^ may influence ASD and ADHD in ways that the use of a single ASM does not, and (3) use of multiple ASMs in pregnancy likely reflects, in part, severity of the underlying indication (e.g., lack of seizure control); therefore, restriction to children born to women reporting ASM monotherapy in pregnancy should reduce the influence of confounding by severity.

We conducted 3 sensitivity analyses using reported use of any ASM as our exposure to test the robustness of findings. First, we removed children whose mothers reported use of the benzodiazepines prescribed in epilepsy (i.e., clonazepam, clobazam) because these medications have different properties (e.g., indications for use, pharmacology) from other ASMs.^3,42^ Second, we estimated models using a minimum age at ASD and ADHD diagnosis of 4 years to capture more stable diagnoses and to allow full coverage in outpatient records. Third, we restricted the analysis to children whose mother had a recorded diagnosis of epilepsy within the 10 years before birth to indicate an active condition.^43^ For the last analysis, we examined associations among all exposure groups to examine whether findings differed according to individual AEDs. All analyses were performed with SAS 9.4 (SAS Institute Inc, Cary, NC).

## Results

Associations between maternal-reported ASMs and ASD and ADHD in children of women with epilepsy are presented in table 2. In this sample, children whose mothers reported use of any ASM (n = 3,316) had elevated risk of ASD (hazard ratio [HR] 1.79, 95% confidence interval [CI] 1.39–2.30) and, to a lesser extent, ADHD (HR 1.27, 95% CI 1.06 = 1.52). These associations were largely unchanged by confounder adjustment, whereas restriction to children born to women who reported monotherapy attenuated the association for ASD (HR 1.52, 95% CI 1.12–2.07) but not ADHD (HR 1.27, 95% CI 1.02–1.58).

**Table 2:**
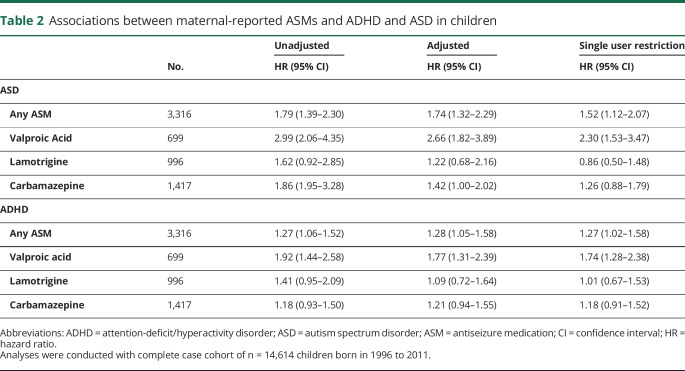
Associations between maternal-reported ASMs and ADHD and ASD in children

Consideration of specific ASMs revealed more pronounced associations with valproic acid (n = 699, 4.78%). The initially observed nearly 3- and 2-fold elevated risks for ASD (95% CI 2.06–4.35) and ADHD (95% CI 1.44–2.58), respectively, were gradually attenuated by adjustment for confounding. In the fully adjusted comparisons restricted to monotherapy, children whose mothers reported use of valproic acid still had a 2.3-fold elevated risk of ASD (95% CI 1.53–3.47) and a 1.7-fold elevated risk of ADHD (95% CI 1.28–2.38) compared to children whose mothers reported no use of ASMs. Notably, of the top 3 ASMs, valproic acid was the least commonly reported use across the study period.

A different pattern emerged for associations with lamotrigine (n = 996, 6.82%), the second most commonly reported ASM. Compared with valproic acid, maternal-reported lamotrigine use was less strongly associated with ASD (HR 1.62, 95% CI 0.92–2.85) and ADHD (HR 1.41, 95% CI 0.95–2.09) at baseline, and these estimates were completely attenuated with adjustment for covariates. Among monotherapy users, lamotrigine was not associated with any elevated risk of ASD (HR 0.66, 95% CI 0.27–1.58) or ADHD (HR 1.00, 95% CI 0.59–1.69).

Finally, for the most commonly reported ASM in the study period, carbamazepine (n = 1,417, 9.7%), yet another pattern of associations emerged. An observed elevated risk of ASD (HR 1.86, 95% CI 1.95–3.28) was substantially attenuated by confounder adjustment (HR 1.42, 95% CI 1.00–2.02) and further restriction to monotherapy (HR 1.26, 95% CI 0.88–1.79). The association with ADHD, on the other hand, was weak and not statistically significant (HR 1.18, 95% CI 0.93–1.50), with estimates largely unaffected by confounding (HR 1.21, 95% CI 0.94–1.55) and the exclusion of polytherapy (HR 1.18, 95% CI 0.91–1.52).

Sensitivity analyses (table 3 and table e-3, doi.org/10.5061/dryad.2z34tmpk0) showed that excluding benzodiazepines from the exposure definition, altering the required minimum age at diagnosis of ASD and ADHD to 4 years of age, and restricting the sample to children born to women with epilepsy diagnoses in the 10 years before childbirth did not change our primary findings (regarding any ASM).

**Table 3:**
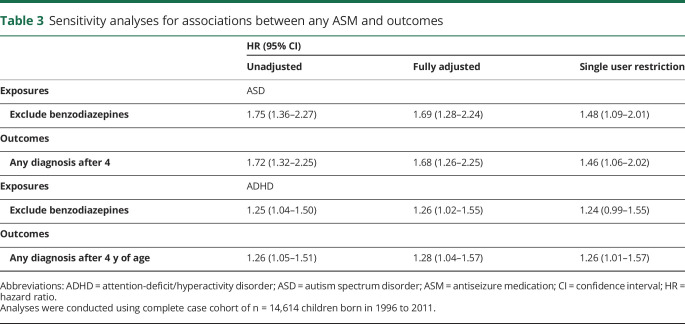
Sensitivity analyses for associations between any ASM and outcomes

## Discussion

In the largest sample to date and with adjustment for a range of pregnancy-related, maternal, and paternal characteristics, we observed associations between maternal-reported ASM use in pregnancy and elevated risks of ASD and ADHD in children. However, examination of specific ASM types revealed diverging findings, highlighting the importance of research on individual drugs in that our results supported the hypothesis that certain ASMs may be associated with greater risk to fetal development.

We found that after adjustment for many important covariates for which previous research has not accounted (e.g., parental psychiatric problems, seizure/epilepsy severity), maternal-reported use of valproic acid, in particular, was associated with increased risk of ASD and ADHD in children. These associations remained after we restricted to women who reported that they used only valproic acid in pregnancy, which suggests that the use of other ASMs did not substantially affect these findings.

In contrast, although children born to women with epilepsy who reported use of lamotrigine in pregnancy had an elevated risk of ASD and ADHD, we observed no evidence of increased risk of ASD and ADHD specifically related to lamotrigine; rather, associations were explained entirely by confounding factors. Although there have been discrepant findings in the literature regarding lamotrigine use in pregnancy and the occurrence of cleft lip/palate in children,for example,^44,45,^ a large majority of research has also suggested that lamotrigine use in pregnancy does not increase the risk of birth defects.^4^ These findings may provide further reassurance to pregnant women and doctors for the use of lamotrigine in pregnancy.

Reported use of carbamazepine was associated with elevated risk of ASD and ADHD in children in unadjusted models; however, these findings were weaker and could not be differentiated from the null hypothesis (despite the fact that carbamazepine was the most commonly reported drug). Furthermore, although associations with ADHD remained across models, adjustment for confounding and restriction to monotherapy led to substantial attenuation in models examining risk of ASD related to carbamazepine. Thus, this pattern of findings may suggest the possibility of residual confounding rather than a causal effect.

More broadly, it is important to interpret the remaining elevated risk of ASD and ADHD in children with caution. Although the adjusted associations may reflect a causal influence, we were not able to rule out all sources of confounding in this study. Clinical recommendations strongly discourage the use of valproic acid for women of childbearing years unless absolutely necessary for the management of seizures because of its association with birth complications (e.g., birth defects).^4,46^ Thus, although findings may be consistent with a causal effect, the independent associations could be confounded by the severity of epilepsy or other indications of use for which our study was not able to account. Although we adjusted for severity in our study by including inpatient hospitalizations for seizures as a covariate and excluding those exposed to ASM polytherapy in final models, it is likely that at least some residual confounding remains.

It is also possible that epilepsy subtype may confound associations because valproic acid, lamotrigine, and carbamazepine do not have the same exact indications for use.^3^ Whereas valproic acid is considered the first-line treatment for generalized epilepsy, lamotrigine is often used when valproic acid is contraindicated (e.g., in women of childbearing years, tolerability concerns).^47^ Carbamazepine is also used for either generalized or focal epilepsy but is often not considered the first line of treatment.^46–49^ However, given our pattern of findings, it does not seem likely that epilepsy subtype would be a strong confounding influence. Specifically, valproic acid and lamotrigine have the most similar indications for use, and whereas valproic acid showed the strongest association with the outcomes under study, lamotrigine showed no association at all.

It is also important to consider the confounding influence of other indications for ASM use because all 3 medications can also be used as mood stabilizers (e.g., treatment for bipolar disorder). Although we adjusted for bipolar disorder in the current analyses and restricted our cohort to children born to women with epilepsy to specifically select those with epilepsy as our indication, it is still possible that mood disorder symptoms may confound associations.

Finally, genetic factors may also still confound associations between maternal use of ASM in pregnancy and NDDs in children because research has shown genetic overlap between epilepsy, ASD, and ADHD.^20^ Genetically informed designs such as a sibling comparison are needed to address such confounding; however, given the chronicity of epilepsy and ASM use, we did not observe enough exposure discordance between siblings to make such a comparison.

This study has several strengths. First, we included a large sample, which enabled more precise estimates of the associations between maternal use of ASMs in pregnancy and ASD and ADHD in children than past research (e.g., typical n < 100;^5,7-11^ largest n = 388 exposed to valproic acid and 647 exposed to lamotrigine^6^). Second, the rich information available from linking several Swedish national registers enabled improved adjustment for confounding. We were able to consider the influence of ASM indication by restriction to women with epilepsy and further adjusting for potential co-occurring conditions (e.g., bipolar disorder). We were also able to adjust for covariates that previous research has not considered, including maternal and paternal factors (e.g., psychiatric diagnoses), as well as maternal use of additional medications and inpatient hospitalizations for seizures in the year before pregnancy. Third, our use of the filled prescription data to check for agreement with maternal-reported use of ASMs in pregnancy extends prior research^34^ documenting agreement between maternal-reported use of ASMs and filled prescriptions for ASMs. Fourth, previous research has documented high validity of ASD and ADHD diagnoses in the Swedish register data.^38–40^

The results must also be considered in light of several additional limitations. First, there may be concern for survival bias due to our requirement that children reach the age of 2. If the treatment leads to higher risk of early death, the children who survive are less likely to have other risk factors for loss, some of which may also influence risk of ASD and ADHD. In such a scenario, estimates of associations between ASM use in pregnancy and childhood ASD and ADHD would be biased toward the null. However, in our data, there was only 1 stillbirth and 2 deaths before the age of 2 among women who used ASMs during pregnancy, suggesting that early deaths are unlikely to have affected our results. Second, due to the coverage of available information, we were not able to follow up all children through the entire risk period for ASD and ADHD development. It will be important to replicate these findings with longer follow-up, particularly given that certain ASMs have become more and less prevalent over time (figure). Third, we were not able to adjust for parental diagnosis of ASD and ADHD because we have outpatient diagnoses starting in 2001 and prescribed drug information starting in 2005. Given that these disorders are heritable,^20^ this is a likely source of confounding in the present study. Fourth, we were also not able to study associations between other specific ASMs and ASD and ADHD in children because of the rarity of several ASMs in the available data (figure). Fifth, due to the nature of the data sources, our means of identifying conditions such as epilepsy and psychiatric conditions may not have captured all with these conditions. Sixth, our use of Swedish data may also limit the generalizability of findings to other populations, although we have no reason to believe potential mechanisms would vary across countries. Seventh, due to the limitations of register data and reliance on diagnoses, we were not able to examine whether ASM use in pregnancy is associated with (1) symptoms that may be comorbid between ASD and ADHD or (2) a broader construct (e.g., developmental delay) that includes individuals with ASD and/or ADHD symptoms or (3) if associations are unique for each outcome. Eighth, we were not able to investigate how ASM dose affected associations because such information was not available to us. Future research should therefore explore potential dose-response patterns. Ninth, we did not examine the effects of ASM polytherapy (i.e., combinations of ASMs or ASM therapy together with other classes of medications) or switches of ASM medications. Although outside the scope of the present study, future research is needed in this area. Finally, to the extent that the remaining observed associations are causal, future research should also investigate whether birth and other offspring outcomes (e.g., birth defects, preterm birth, small for gestational age, epilepsy) may mediate associations or whether these outcomes are independently related to ASM use in pregnancy.

The present study did not find support for a causal association between maternal use of lamotrigine in pregnancy and ASD and ADHD in children. We observed elevated risk of ASD and ADHD related to maternal use of valproic acid, while associations with carbamazepine were weak and not statistically significant. Although we could not rule out all potential confounding factors, our findings add to a growing body of evidence that suggests that certain ASMs (i.e., lamotrigine) may be safer than others in pregnancy.

## Glossary

ADHD: attention-deficit/hyperactivity disorder
ASD: autism spectrum disorder
ASM: antiseizure medication
ATC: Anatomical Therapeutic Chemical
CI: confidence interval
HR: hazard ratio
ICD: International Classification of Diseases
NDD: neurodevelopmental disorder
NPR: National Patient Register
